# Supplementary material for: Mechanisms of antibiotic action shape the fitness landscapes of resistance mutations
Source: Comput Struct Biotechnol J. 2022 Aug 24;20:4688–703. doi: 10.1016/j.csbj.2022.08.030 (PMC9463365; doi:10.1016/j.csbj.2022.08.030)
Supplement: Supplementary figures and text [file mmc8.docx]

| **Model parameters** | | | | |
| --- | --- | --- | --- | --- |
| **Name** | **Description** | **Value** | **Units** | **Source** |
| *N* | Number of target proteins per cell (i.e. HMM-PBP copy number) | 600 | cell^-1^ | [1] |
| *G_0_* | Bacterial growth rate in the absence of drug | 1.75 | hr^-1^ | Fit by model |
| *G_N_* | Bacterial growth rate in saturating concentrations of drug | 1.75 | hr^-1^ | Fit by model |
| *D_0_* | Bacterial death rate in the absence of drug | 5.40 x 10^-3^ | hr ^-1^ | [2] |
| *D_N_* | Bacterial death rate in saturating concentrations of drug | 29.1 | hr ^-1^ | Fit by model |
| *k_F_* | *β*-lactam acylation rate | 130 | M ^-1^ sec ^-1^ | [3] |
| *k_R_* | Deacylation rate | 1 x 10^-4^ | sec ^-1^ | [3] |
| *α_G_* | Steepness of growth rate function G[*i*] | n/a | # drug-target complexes^-1^ | n/a |
| *α_D_* | Steepness of death rate function D[*i*] | 7.67 | # drug-target complexes^-1^ | Fit by model |
| *γ_G_* | Inflection point of growth rate function G[*i*] | n/a | # drug-target complexes | n/a |
| *γ_D_* | Inflection point of death rate function D[*i*] | 1310 | # drug-target complexes | Fit by model |
| *B* | Initial size of bacterial population at the start of drug treatment | Varies | cell ml^-1^ | n/a |
| *µ_R_* | Mutation rate for drug resistance emergence | 2.00 x 10^-7^ | cell^-1^ division^-1^ | [4, 5] |
| *µ_C_* | Mutation rate for emergence of secondary mutations in resistant strains | 2.00 x 10^-6^ | cell^-1^ division^-1^ | [4, 5] |
| *c_R_* | Cost of resistance mutation, such that the antibiotic-free growth rate of a resistant mutant is *G_0_* (1 - *c_R_*) | 0.25 | Non-dimensional | [6] |

**Supplementary Table S1 – Model parameterization to ampicillin time-kill curves.**
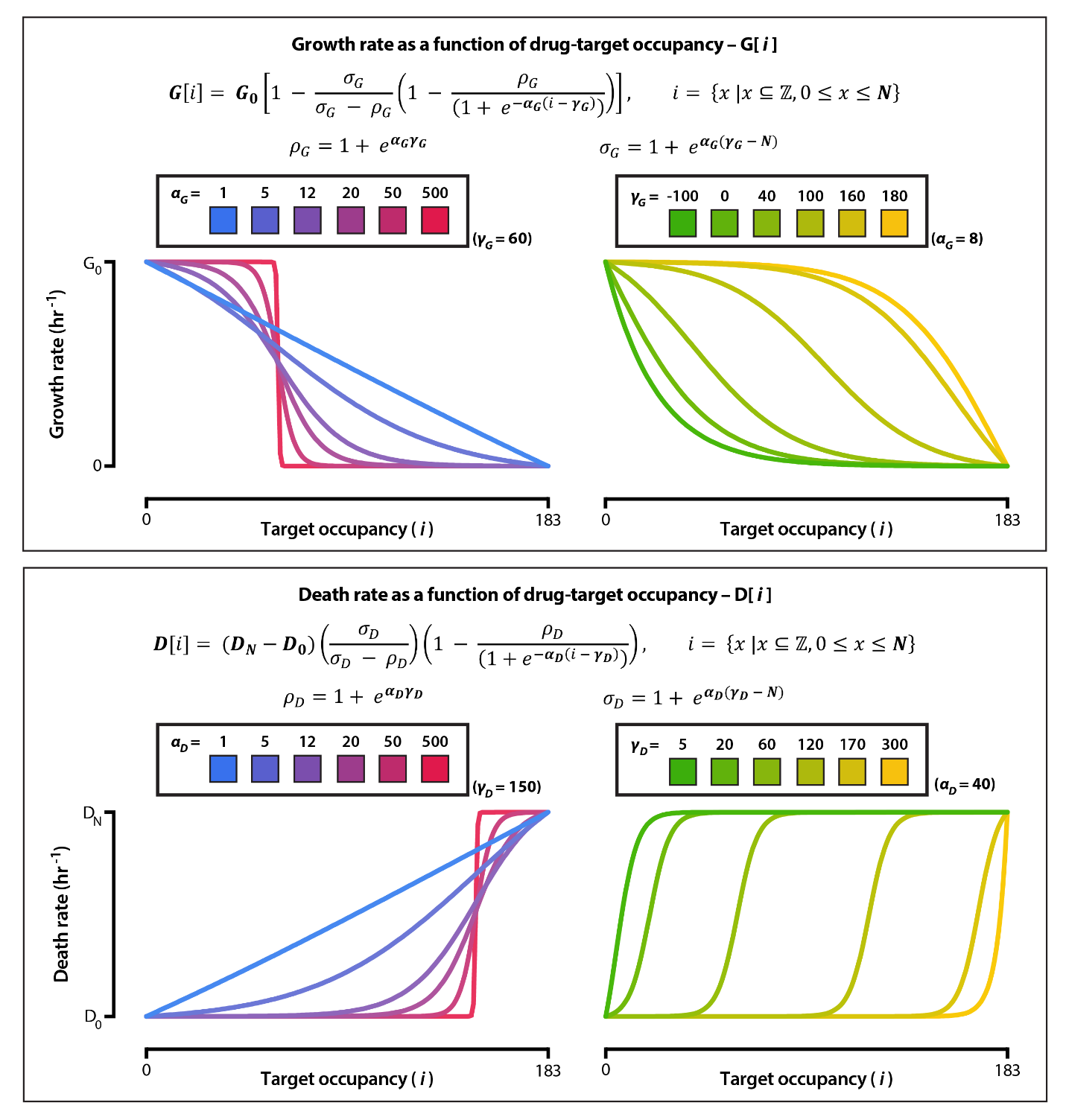


**Supplementary Figure S1 – Bacterial growth and death rates as a function of drug-target occupancy.** We define the functions G[*i*] and D[*i*] as constrained logistic curves such that G[*i* = 0] = *G_o_*, G[*i* = *N*] = 0, D[*i* = 0] = *D_0_*, and D[*i* = *N*] = *D_N_*. The parameters *α_G_* and *α_D_* define the steepness of the logistic curves for the growth and death rate function, respectively. *α_G_* and *α_D_* are unitless and range from 1 to 500; 1 yields a quasi-linear function, while 500 yields a quasi-step function. The parameters *γ_G_* and *γ_D_* define the inflection point of the logistic curves for the growth and death rate function, respectively. *γ_G_* ranges from –*N* to *N* and *γ_D_* ranges from 0 to 2*N*; the curve is quasi-sigmoidal if *γ_G_* and *γ_D_* are in between 0 and N and is quasi-exponential if *γ_G_* and *γ_D_* are outside of these bounds.

**
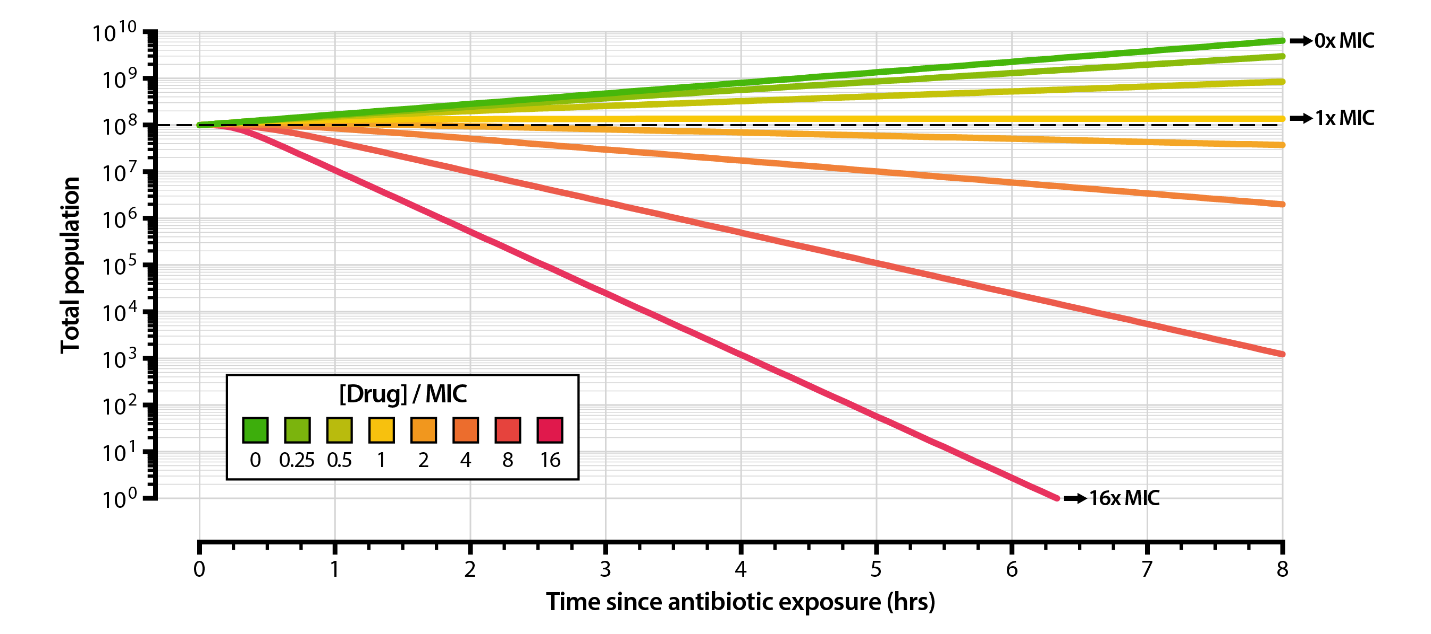
**

**Supplementary Figure S2 – Simulated time-kill curves of *Escherichia coli* exposed to a range of drug concentrations.** We used the parameter set outlined in **Table 1** to model the growth and death of bacterial populations subjected to drug concentrations up to 16x minimum inhibitory concentration (MIC). Drug concentrations are expressed as factors of the MIC. The net growth rate of the entire bacterial population over the time course of the simulation decreases with increasing drug concentration.

**
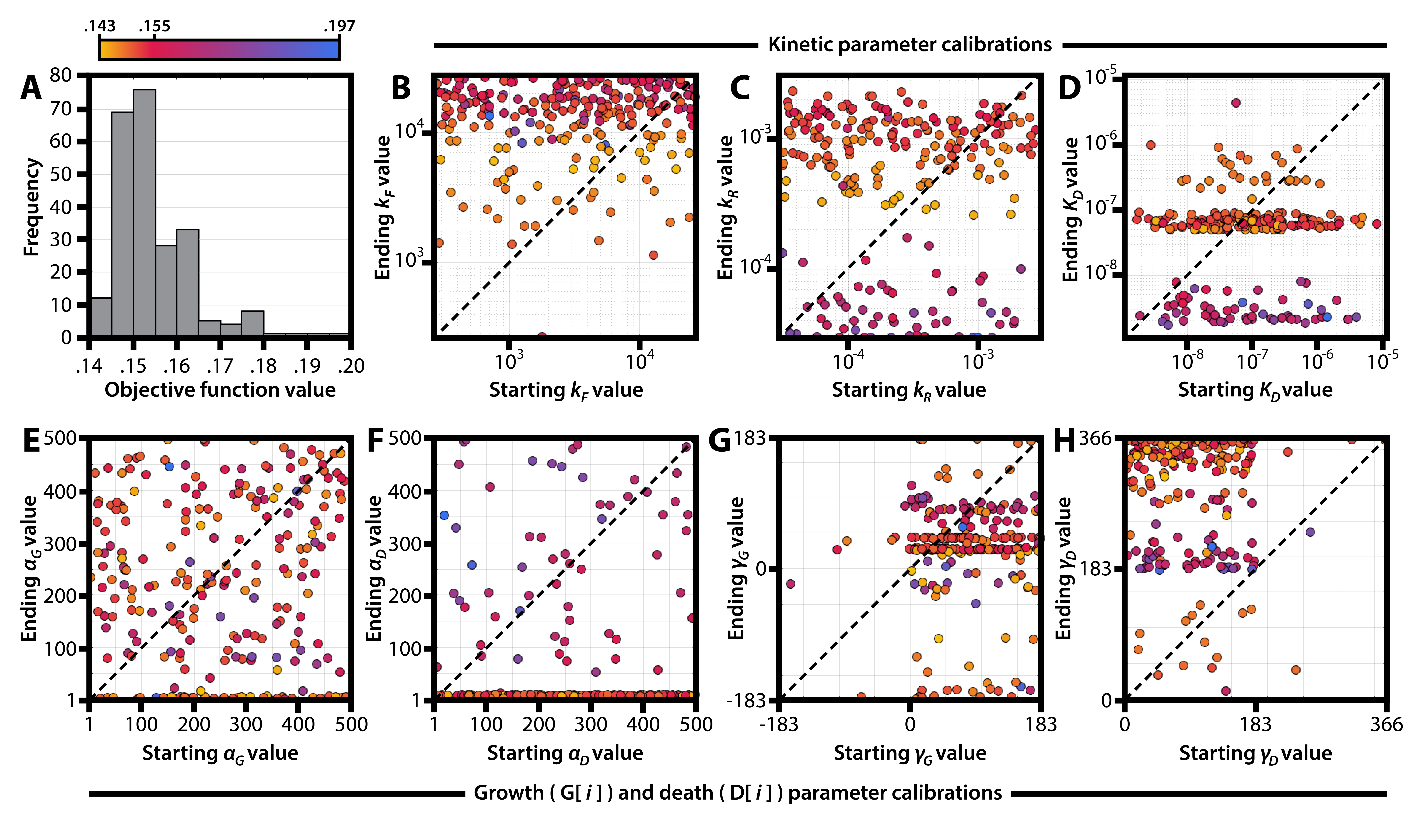
**

**Supplementary Figure S3 – Results from 249 independent model calibrations to experimental data.** We used adaptive simulated annealing coupled with gradient descent (see **Methods**, *Model calibration via simulated annealing*) to fit the model to experimental kill curve data of *E. coli* exposed to ciprofloxacin (**Supporting Data File S1**). Shown in this figure are the results for 249 independent model fits (**Supporting Data File S3**), each beginning with randomly-chosen values for the parameters describing drug-target binding rate *k_F_*, drug-target unbinding rate *k_R_*, steepness of the growth rate function *α_G_*, steepness of the death rate function *α_D_*, inflection point of the growth rate function *γ_G_*, and inflection point of the death rate function *γ_D_*. (**A**) Frequency distribution of objective function values obtained from independent model calibrations. The objective function value describes the goodness of the fit between experimental data and simulation; smaller values indicate higher goodness of fit. (**B-H**) Optimization plots showing randomly chosen initial parameter values (x-axis) and calibrated parameter values (y-axis) for all independent model calibrations. The optimized parameters are *k_F_* (**B**), *k_R_* (**C**), *K_D_* (the ratio of *k_R_* to *k_F_*) (**D**), *α_G_* (**E**), *α_D_* (**F**), *γ_G_* (**G**), and *γ_D_* (**H**). The final objective function value of each model fit is colored according to the color bar above panel (**A**).

**
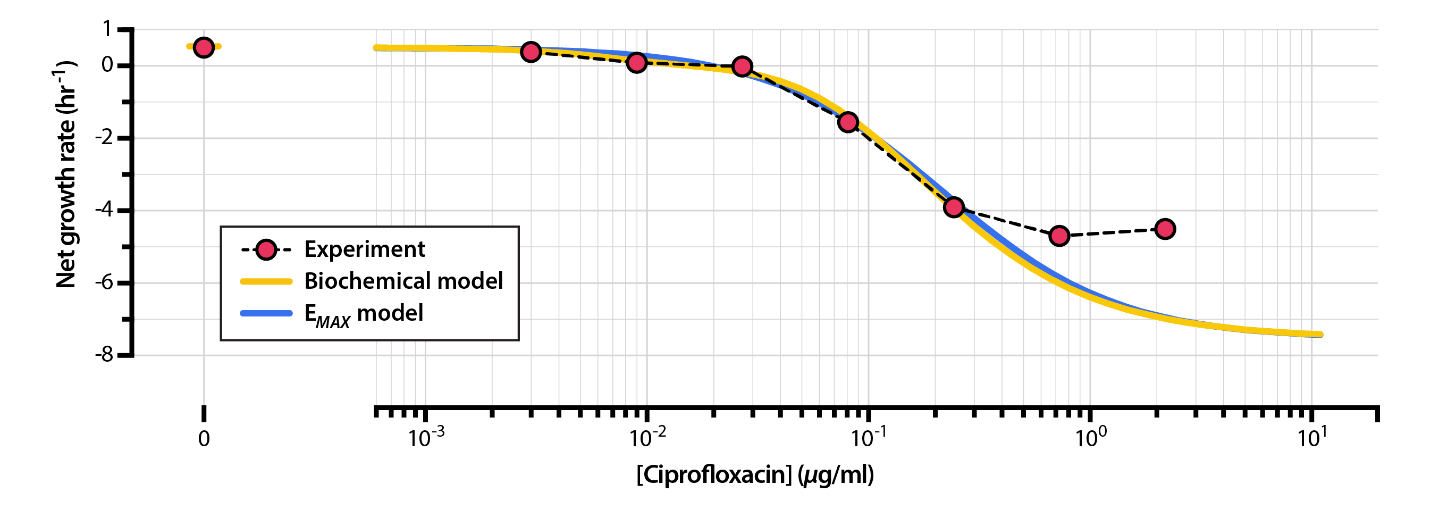
**

**Supplementary Figure S4 – Pharmacodynamic curves generated from experimental data and from the calibrated model.** The experimental pharmacodynamic curve was generated by calculating the net growth rates of *E. coli* exposed to a set of ciprofloxacin drug concentrations (**Supporting Data File S1**). The time-kill curves of this same experimental dataset are shown in **Figure 2A**; see **Supporting Data File S4** for experimental data on net growth rate as a function of drug concentration. The model-calibrated pharmacodynamic curve was generated by simulating bacterial time-kill curves over the same range of drug concentrations used in the experiment and calculating associated net growth rates.

**
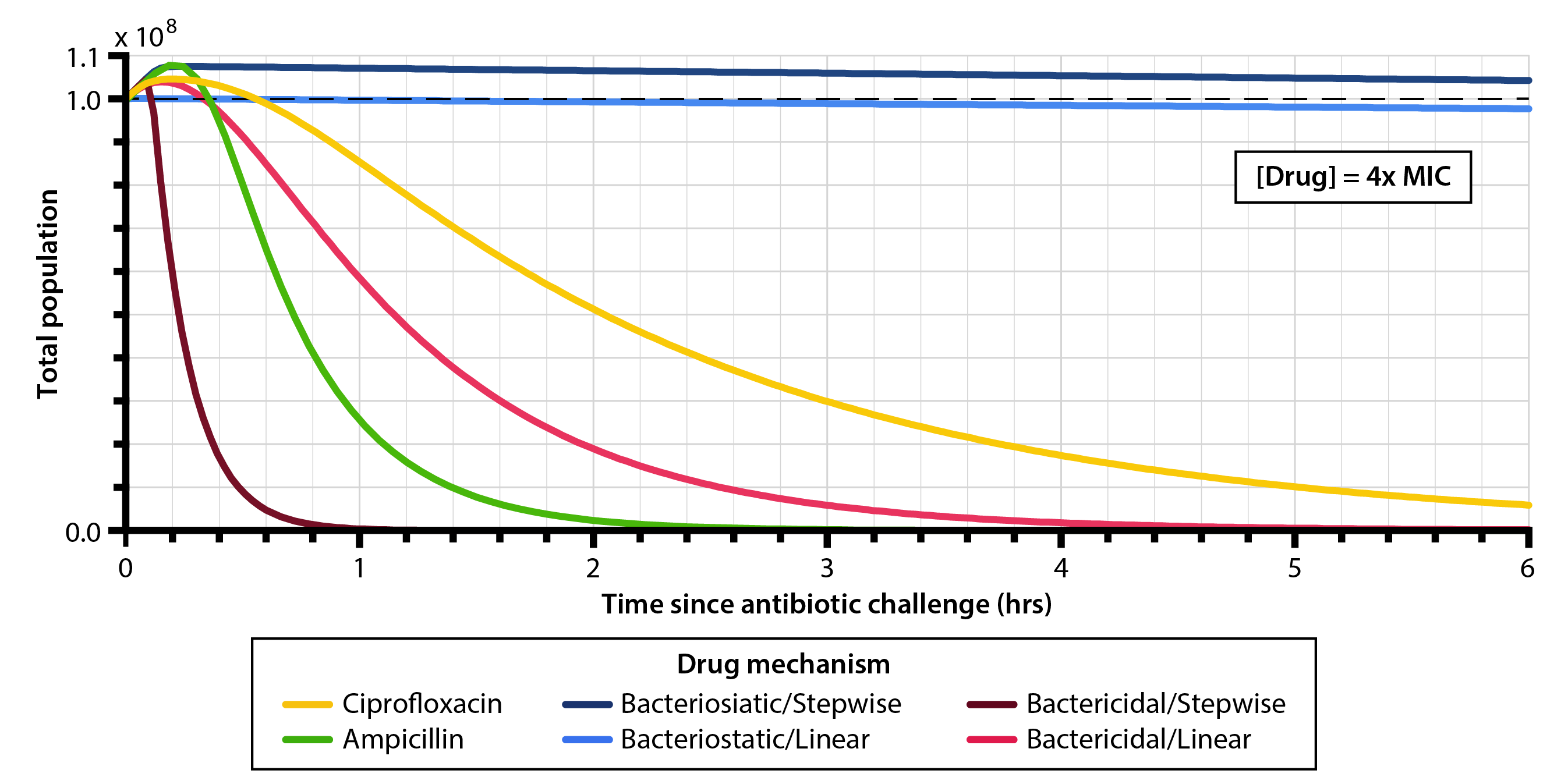
**

**Supplementary Figure S5 – Simulated population curves for ciprofloxacin and for four extreme modes of antibiotic drug mechanism.** We simulated a bacterial population of 10^8^ cells exposed to antibiotic drug at 4x MIC. The ciprofloxacin curve corresponds to the drug mechanism obtained from the model calibration to experimental data and detailed in **Figure 2C**, and the remaining curves correspond to the extreme schemes of drug mechanism shown in **Figure 2D**.

**
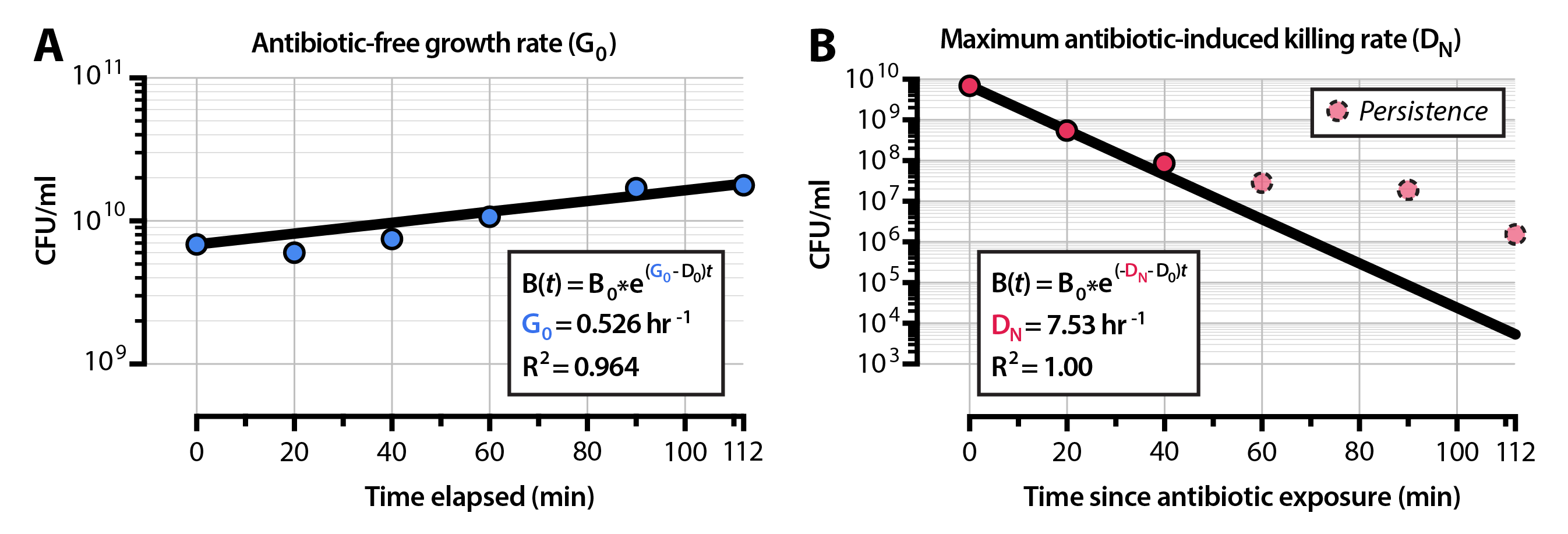
**

**Supplementary Figure S6 – Obtaining *G_0_* and *D_N_* from experimental data.** (**A**) To obtain the value of *G_0_* (growth rate in the absence of antibiotic) used in simulations, we fit an exponential growth curve to experimental data for *E. coli* cells grown in the absence of antibiotic. (**B**) To determine the value of *D_N_* (maximum death rate in saturating conditions of antibiotic), we fit an exponential decay curve to experimental data for *E. coli* cells exposed to 2.19 µg/ml of ciprofloxacin (~200 x MIC). The population size deviates from exponential decay at later timepoints (dashed and shaded) likely because of the emergence of persistent subpopulations of bacteria [7]. The R^2^ values shown are the linear correlation coefficients for the model fit, and are not the correlation coefficients for the log-transform of the data.

**
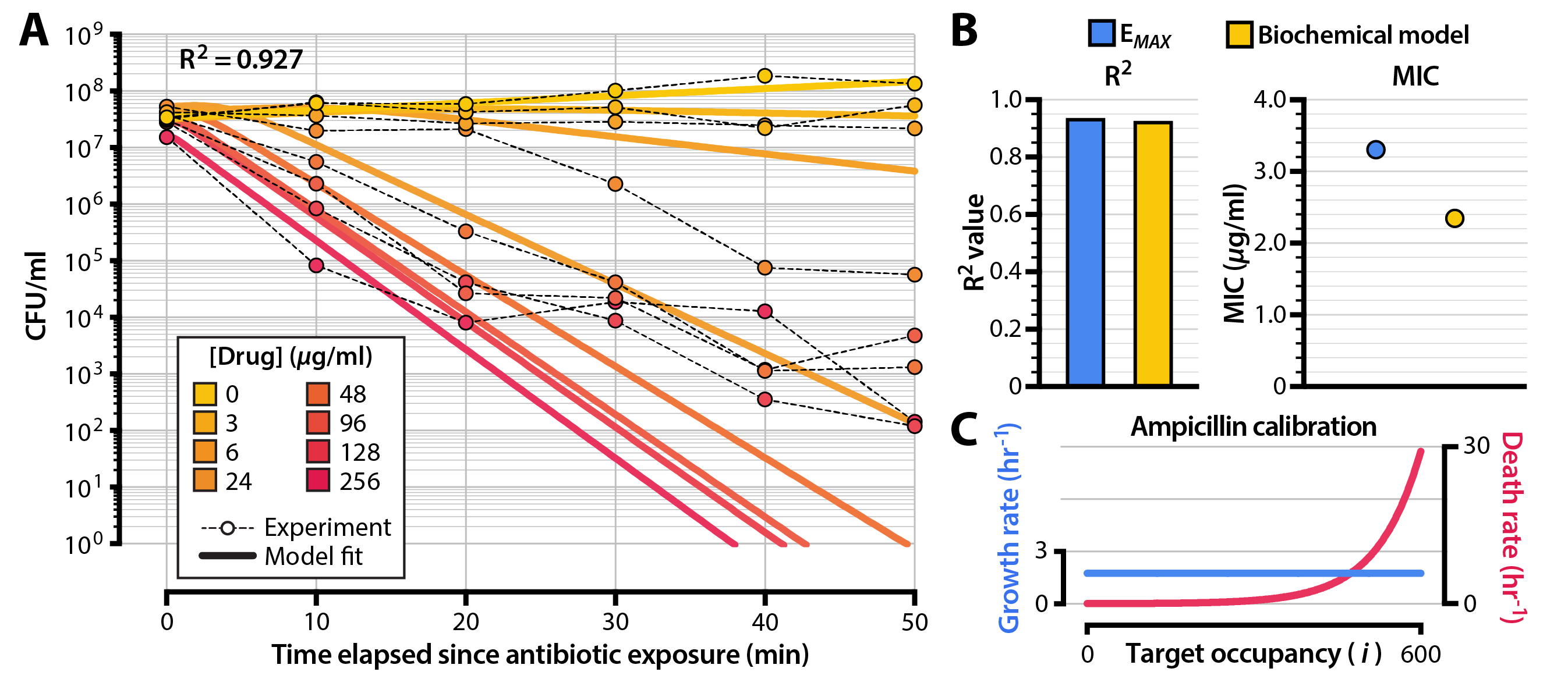
**

**Supplementary Figure S7 – Model calibration to ampicillin time-kill curves.** (**A**) Comparison between calibrated biochemical model (solid lines) and experimental data (shaded points). The experimental data represent time-kill curves of *Escherichia coli* exposed to ampicillin. (**B**) Comparison of the calibrated biochemical model with the *E_MAX_* pharmacodynamic model [8]. We fit the *E_MAX_* model to the same experimental dataset shown in panel (A) and compared Pearson correlation coefficients (R^2^) and MICs. (**C**) Cellular growth and death rates as a function of acylated PBP number (*i*) for the model calibrated to the experimental data shown in panel (A). Values for parameters of the calibrated model are given in **Supplementary Table S1**.

**
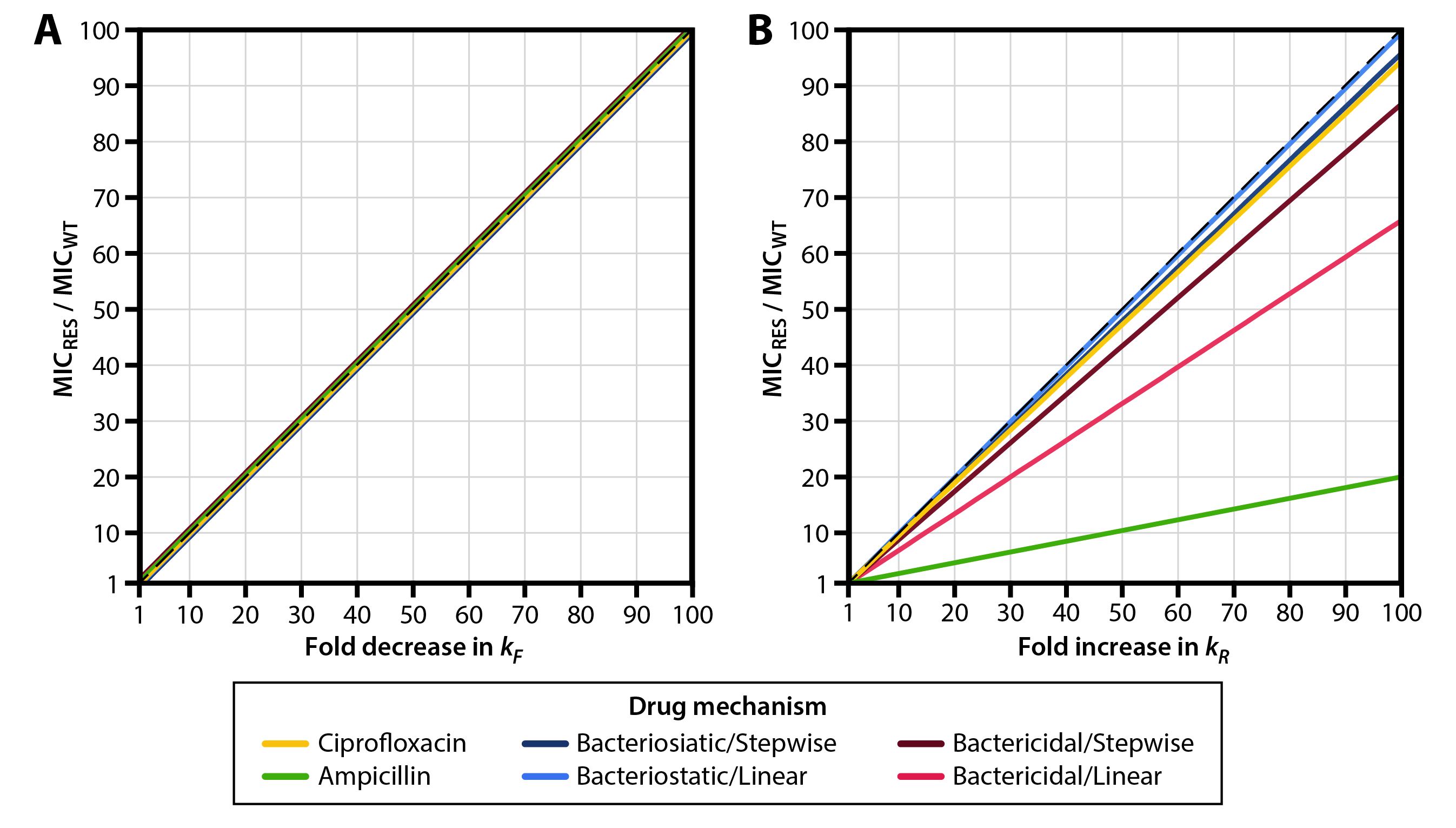
**

**Supplementary Figure S8 – MIC as a function of drug-target binding and unbinding kinetics.** The MIC of a mutant (normalized to the MIC of the wild-type) is plotted against the fold-change in (**A**) drug-target binding (*k_F_*) or (**B**) drug-target complex disassociation (*k_R_*). For this simulation, mutants have no fitness costs associated with changes in *k_F_* and *k_R_* (*c_R_* = 0). For drug-target binding (*k_F_*), fold increase in MIC is directly proportional to fold decrease in *k_F_* for all drug mechanisms. In both panels, the dashed line indicates the line of direct proportionality. MIC_WT_: MIC of the wild-type strain; MIC_RES_: MIC of the resistant strain.

**
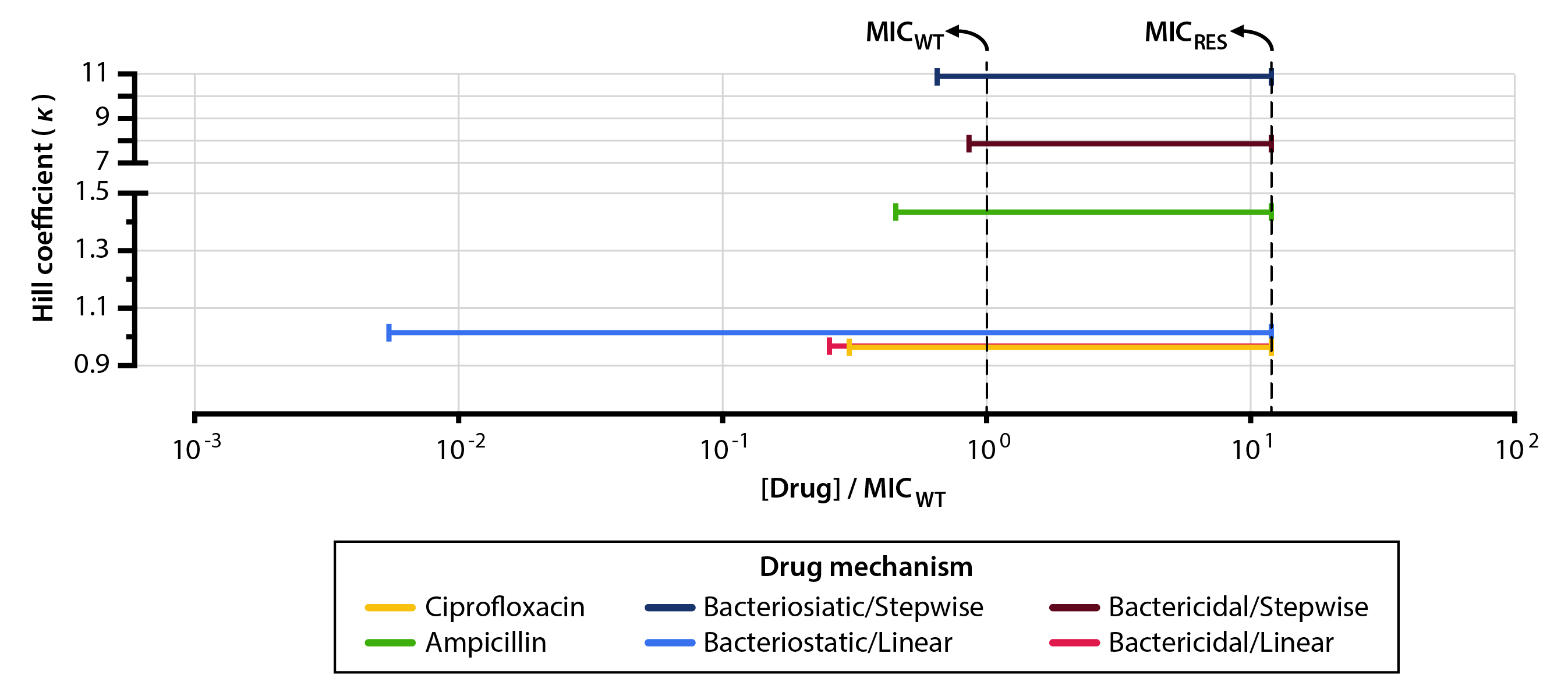
**

**Supplementary Figure S9 – Drugs with steeper pharmacodynamic curves have narrower resistance selection windows given a cellular effect (bacteriostatic/bactericidal).** To quantify the steepness of pharmacodynamic curves, we fit the curves for drug-resistant strains shown in **Figure 4C** to the pharmacodynamic function formulated by Regoes et al. [8]. The equation describes the net growth rate *G_net_* of a bacterial population as a function of drug concentration *C_0_* and other parameters (MIC, *G_0_*, *D_N_*) derived from the model:

$$G_{net}= G_{0}- \frac{(G_{0}-D_{N}){(\frac{C_{0}}{\mathrm{MIC}})}^{\kappa}}{{(\frac{C_{0}}{\mathrm{MIC}})}^{\kappa}-(\frac{D_{N}}{G_{0}})}$$

In this equation, *κ* describes the Hill coefficient, which serves as a measure of the steepness of the pharmacodynamic curve. Larger values of *κ* indicate steeper curves. For each of the drug mechanisms described in this study (**Supplementary File S2**), we generated pharmacodynamic curves for drug-resistant mutants (**Figure 4C**, solid lines), determined the value of *κ* that best fits the curve, and plotted *κ* against the range of drug concentrations that represents the resistance selection window (**Figure 4D**). MIC_WT_: MIC of the wild-type strain; MIC_RES_: MIC of the resistant strain.

**
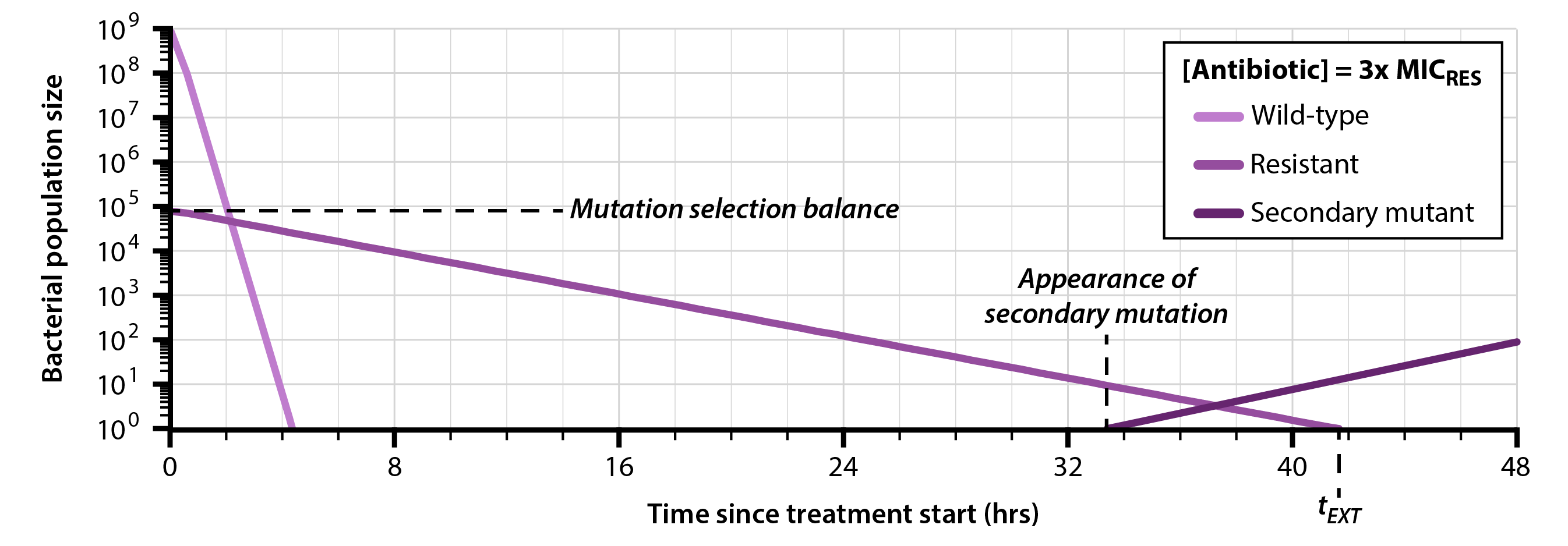
**

**Supplementary Figure S10 – Emergence of secondary mutations within subpopulations of drug-resistant bacteria during antibiotic treatment.** When simulating the emergence of secondary mutations, we assume that a drug-resistant subpopulation (middle purple) of bacteria is present at the start of treatment; the size of this subpopulation is given by the mutation selection balance of the allele that confers the drug-resistance phenotype [9]. We calculate the probability that a drug-resistant strain with secondary mutations (dark purple) emerges from this subpopulation before the elimination of the drug-resistant strain (at time *t_EXT_*).

**
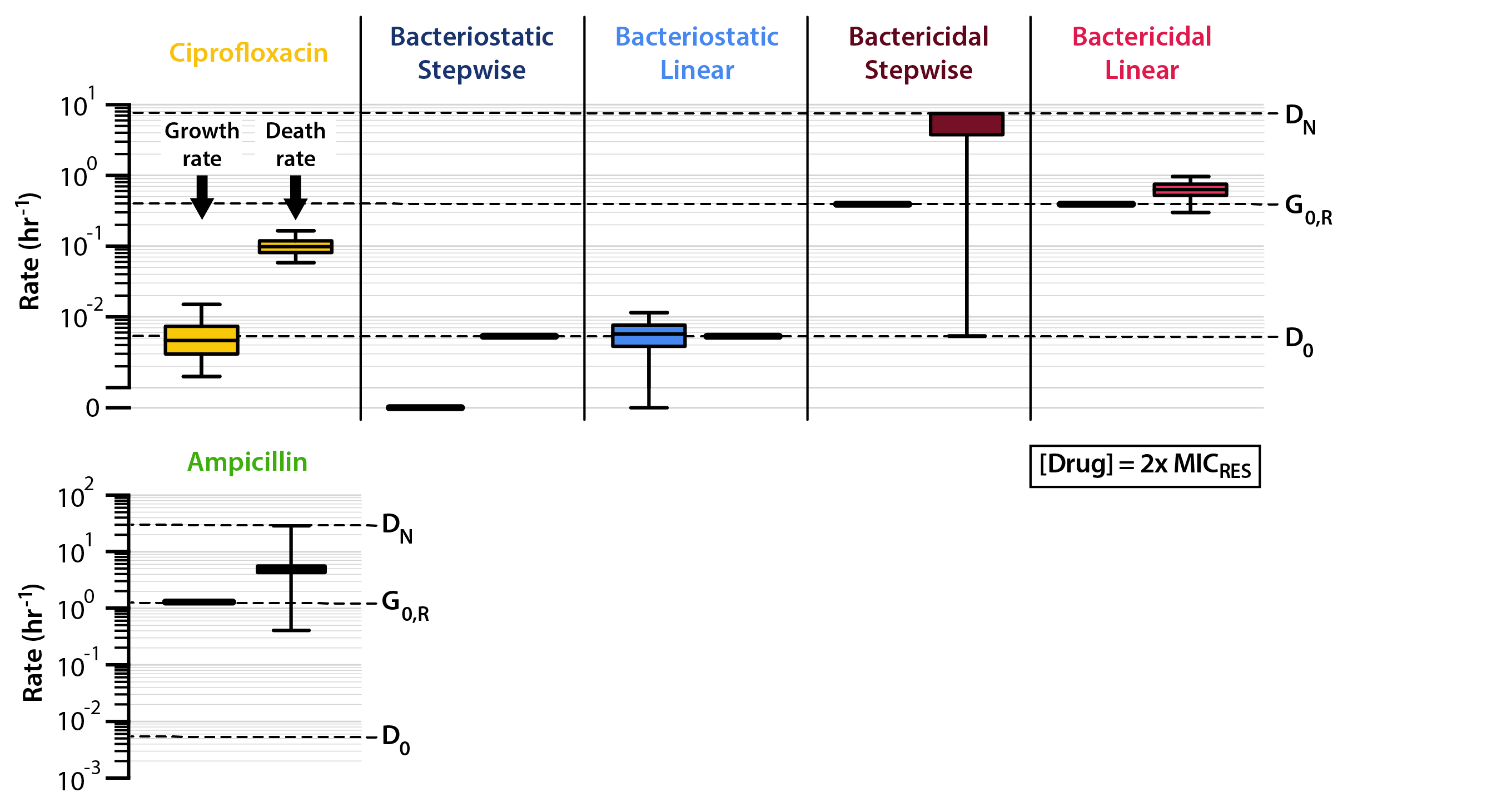
**

**Supplementary Figure S11 – Distributions of growth and death rates for drug-resistant bacterial subpopulations undergoing steady-state exponential decline at 2x MIC_RES_.** Boxes denote the central 50% of the growth and death rate distributions, and whiskers denote the central 95% of the growth and death rate distributions.

**
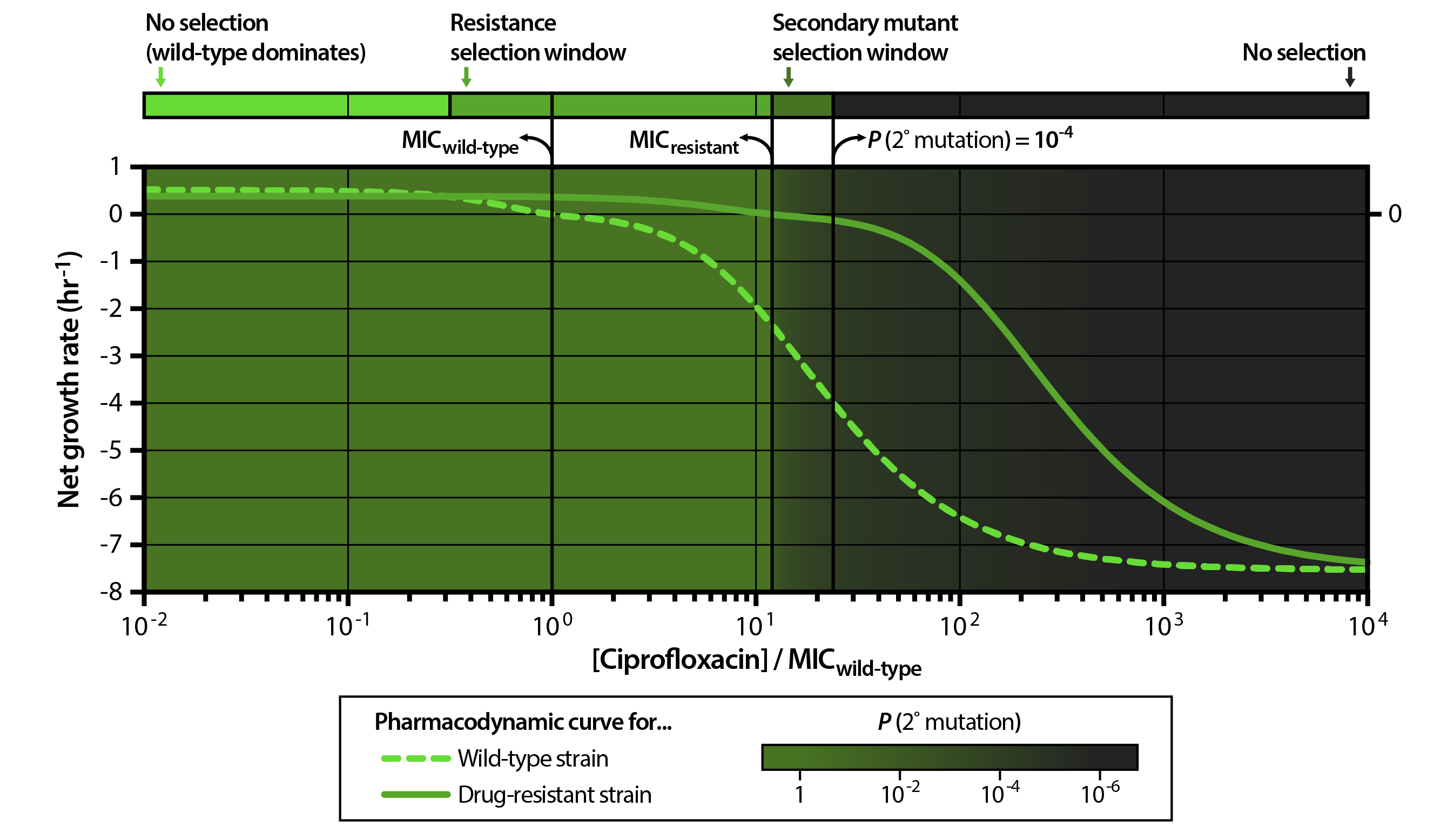
**

**Supplementary Figure S12 – The secondary mutant selection window.** The secondary mutant selection window comprises the drug concentration range over which the net growth of the drug-resistant strain is negative but the probability of secondary resistance emergence before the end of treatment exceeds a defined threshold (in our simulations, 10^-4^, or a 1 in 10,000 chance). Four regimes of selection exist: the null selection window in which the wild-type strain dominates, the resistance selection window, the secondary mutant selection window, and the complete killing window. We simplify these four regimes by disregarding the relative strengths of selection for each strain in each regime and we instead illustrate the boundaries of each region along a drug concentration axis (top bar); these simplified selection regimes are shown for all six drug mechanisms studied in **Figure 5E**.

**
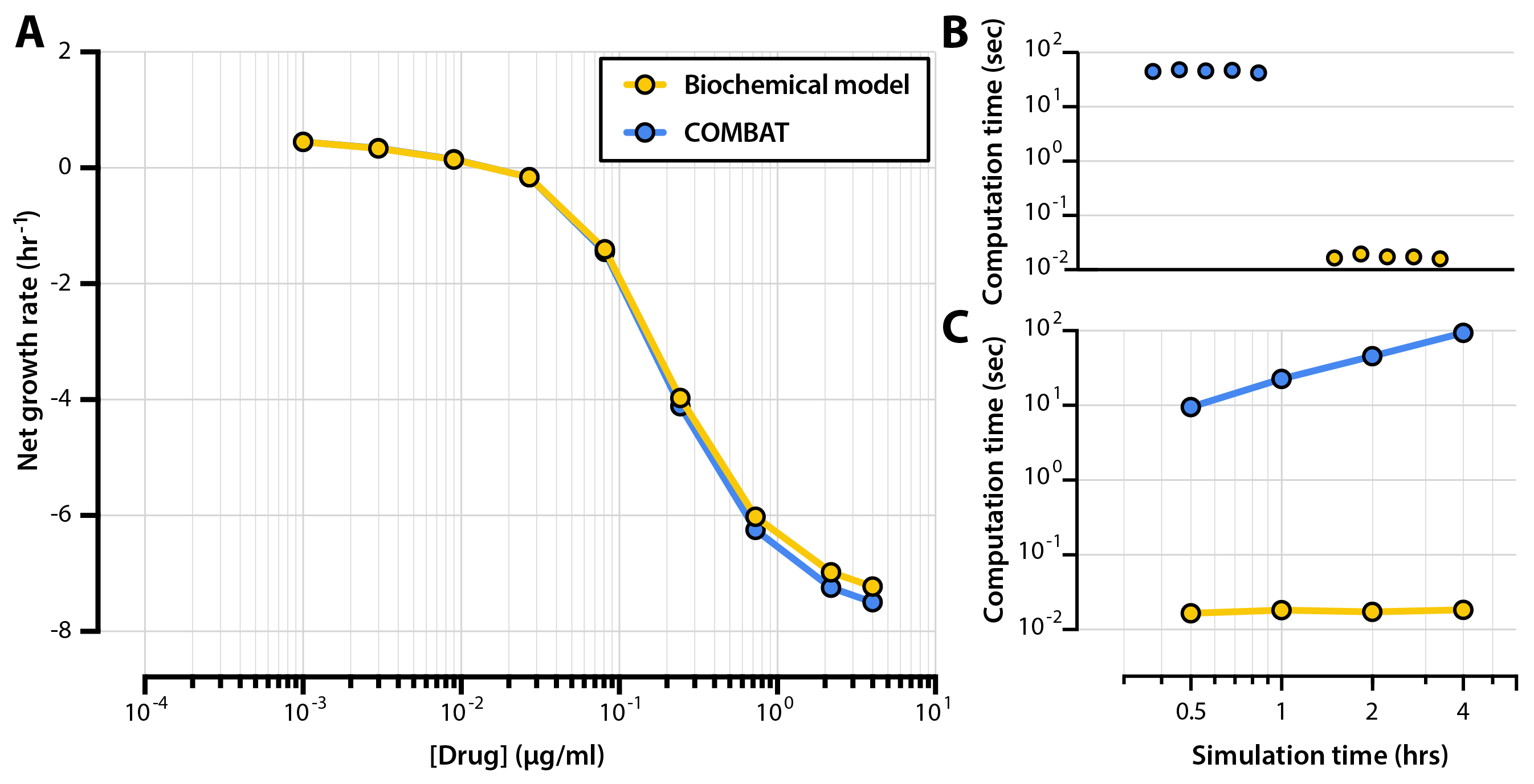
**

**Supplementary Figure S13 – Comparison of biochemical model with COMBAT.** (**A**) Pharmacodynamic curves generated using the linear biochemical model reported in this study and COMBAT, a nonlinear formulation reported previously [10]. Net growth rate was calculated using a simulation time of two hours. (**B**) Time taken to compute the pharmacodynamic curves shown in panel (A) using the linear biochemical model reported in this study and COMBAT. Simulations were repeated five times using each model. **(C**) Time taken to compute pharmacodynamic curves using different simulation times ranging from 30 minutes to two hours. Data shown are the mean of five independent runs of each model. All simulations were conducted on a 2020 Apple MacBook Pro™️ using a 2.3 GHz Intel Quad-Core i7™️ processor with 32 GB of memory.

**Supplementary text**

*The simulated annealing algorithm*

Simulated annealing gets its name because it resembles the physical process of the annealing of solids or oligos in solution. The experimental procedure for annealing oligos involves heating a solution containing the oligos, then slowly cooling it. The reason this is done is to allow the oligos to sample different states. At higher temperatures, the oligos can interact in higher energy configurations; at lower temperatures, the oligos become “trapped” into lower energy (that is, properly annealed) states because there is insufficient energy within the system to allow them to escape those energy minima.

Simulated annealing works by the same principle. Instead of two oligos in solution, we have a matrix of numbers that represent experimental data and a matrix of numbers that represent model predictions. The goal is to minimize the difference between the experimental data and the model predictions (that is, to minimize the “energy” between these matrices). With every iteration, the simulated annealing algorithm randomly chooses a new set of model parameters and calculates the energy between the model prediction with these new parameters and the experimental data. If the energy is lower than it was at the previous iteration (that is, the fit between the model and the data is better), the algorithm accepts this new set of parameters. If the energy is higher, the algorithm may accept this new set of parameters with some probability that is controlled by the “temperature” of the system. Just as with oligo annealing, as the temperature goes down, so the probability that the system transitions to a higher energy state also goes down. By slowly lowering the temperature with each successive iteration of the algorithm, we gradually “trap” the model into a set of parameter values that minimizes the difference between the experimental data and the model predictions.

Because the algorithm can accept higher energy states (that is, worse model fits) with some probability, simulated annealing is capable of escaping local minima and is therefore considered a global optimization method. This means it is, in theory, capable of finding the absolute best fit of the model to the experimental data, regardless of the initial parameter values it is given. In practice, however, this can take an enormous amount of time, so we run the algorithm many (>100) times for shorter periods (4000 iterations each time) from different (random) starting parameter sets to see if the algorithm consistently converges onto a common set of parameter values. Simulated annealing is an excellent tool for fitting models with a medium to large number of parameters. When fitting our model to ciprofloxacin time-kill curve data, for example, we use simulated annealing to fit six parameters.

*On the convergence of model parameters*

We leverage simulated annealing, a stochastic global optimization method, to fit our model to experimental time-kill curve data. Because the method is stochastic, not all replicate runs of the optimization protocol lead to identical minima. The parameters reported in **Table 1** and illustrated in **Figures 2A-C** are derived from the optimization run that yielded the lowest objective function value of 249 independent runs. Performing numerous independent runs to explore parameter comprehensively is a technique similar to parallel simulated annealing, which has been described previously [11]. **Supplementary Figure S3** shows that, for optimization runs with the smallest objective function values (i.e. the best fits, indicated by yellow points), the values of *k_F_* (**Supplementary Figure S3B**) and *k_R_* (**Supplementary Figure S3C**) converge consistently (to approximately 5 x 10^3^ M^-1^ sec^-1^ for *k_F_* and 3 x 10^-4^ sec^-1^ for *k_R_*). Because *k_F_* and *k_R_* converge consistently, *K_D_* also converges.

The parameters *𝝰_G_* and *𝛄_G_*, which control the steepness and inflection point of the growth rate curve as a function of target occupancy, do not appear to converge (**Supplementary Figure S3F**), suggesting that these parameters have little influence on data fitting. This is likely because growth rates are constrained to a narrow range of values (from 0 to 0.526 hr^-1^). Bacterial death rates, however, can take on a wider range of values (from 0 to 7.53 hr^-1^) and thus exert a stronger influence on data fitting. Consequently, for optimization runs with low objective function values, the parameter *𝝰_D_* does consistently converge to low values (**Supplementary Figure S3F**). The associated inflection point parameter *𝛄_D_* shows little evidence of convergence. This is likely a side effect of the associated value of *𝝰_D_*—a low value for *𝝰_D_* indicates a curve that is more linear as opposed to stepwise (**Supplementary Figure S1**). For more linear curves, the value of the inflection point is thus of lesser importance.

*Comparison between the model reported in this study and COMBAT*

The model described in this study is a linear case of COMBAT, a nonlinear formulation that has been described previously [10]. We find that the linear model and COMBAT generate similar pharmacodynamic curves when given identical parameter sets, but that the linear model reported in this study can simulate pharmacodynamic curves >1000 times more rapidly than can COMBAT (**Supplementary Figure S13**). Additionally, both the linear model described in this study and COMBAT predict similar death rate functions when they are fit to separate experimental replicates of ampicillin time-kill curve data (compare **Supplementary Figure S7C** of our study to Figure 5B of [10]). This suggests that both models can derive similar pharmacodynamic insights from experimental data. However, we note that growth and death rate functions fit to experimental data may not be identical between the models because COMBAT uses exponential functions to describe growth and death rate as a function of target occupancy whereas our linear model uses a constrained logistic function (**Supplementary Figure S1**).

Using both models, generating pharmacodynamic curves requires simulating bacterial populations under antibiotic exposure for some predefined amount of time and calculating a net growth rate. The computational speed-up of the linear model therefore applies to any task that requires simulating bacterial populations in time. This includes fitting model parameters to experimental time-kill curve data (such as that reported in **Figure 2** and **Supplementary Figure S7**) as well as calculating the time required to bring a bacterial population to extinction under some constant drug concentration (a calculation that is necessary for the derivation of secondary mutant selection windows as reported in **Figure 5E**).

We also describe a strategy for calculating MIC directly from the matrix representation of the linear model (see *Methods Section 4.3*). Such a calculation is not possible using COMBAT, which requires simulating pharmacodynamic curves to calculate MIC. Because of this, the linear model reported in this study is ideally suited for simulations that require the exploration of parameter space at high resolutions, such as those reported in **Figure 3**.

Two limitations of the linear model reported in this study are (1) the inability to model a carrying capacity for the bacterial population and (2) the inability to model differing intra- and extracellular drug concentrations. Modeling a carrying capacity is important if the objective is to simulate bacterial populations for extended periods of time (>1 day) under conditions of very low antibiotic concentration (see, for instance, [12]). A formulation with an ability to model differing intra- and extracellular drug concentrations is crucial for studying certain resistance mechanisms, such as drug efflux (see, for instance, [13]). The linear model described in this study is therefore well suited for fitting parameters to experimental time-kill curve data gathered over short timescales (<2 hours), as is shown in **Figure 2**. However, we would recommend different (nonlinear) formulations for studies that include additional modeling objectives such as and accommodating differing intra- and extracellular drug concentrations.

*On the heterogeneity of bacterial subpopulations*

A major determinant of the heterogeneity of simulated bacterial populations under antibiotic exposure is the magnitude of the bacterial growth rate relative to the rate of drug-target inactivation. Our model assumes that when bacteria divide, they partition their inactivated drug-target complexes between two daughter cells according to a binomial distribution (**Equation 2**). Growth therefore has the effect of diluting inactivated drug-target complexes and placing daughter cells into regimes of lower drug-target occupancy

If a drug acts as a bacteriostatic agent (that is, there exists a region of the drug-target occupancy curve where growth rate is at or near zero) and drug concentration is sufficiently high (~2x MIC or greater), growth rate may be close to zero for the entire bacterial population, so the aforementioned dilution effect is nonexistent. As a consequence of this, drugs like ciprofloxacin generate fairly narrow distributions of bacterial subpopulations under antibiotic exposure (**Figure 5B**, left panel).

If a drug acts purely as a bactericidal agent, then growth rate is unaffected as a function of drug-target occupancy, so the dilution effect does occur. However, **Figure 5B** indicates that the hypothetical bactericidal agents we modeled (light and dark red lines) also have fairly narrow subpopulation distributions. What dictates the width of the bacterial subpopulation distribution under this condition is the magnitude of *k_F_* relative to *G_0_*. If *k_F_* is sufficiently high, then the free drug targets within any nascent daughter cell will immediately become bound to drug. This leads to the narrow distribution seen for the bactericidal agents in the left panel of **Figure 5B**. However, if *k_F_* takes on a smaller value, then drug-target dilution due to cell division competes appreciably with drug-target binding. This is what we observe for the steady-state subpopulation distribution of bacteria exposed to ampicillin (**Figure 5B**, right panel). For ampicillin, *k_F_* is 130 M^-1^s^-1^, whereas it is 5230 M^-1^s^-1^ for ciprofloxacin and the hypothetical antibiotics with purely bactericidal activity (all bacterial populations were modeled to have identical growth rates). Because of the smaller value of *k_F_*, we observe a long tail to the distribution of bacteria exposed to ampicillin. The heterogeneity of bacterial subpopulations becomes an important factor for cells under mutational pressure, as we assume that only dividing cells are capable of mutating. This is likely the basis for our observation that, for a given effect steepness (linear or stepwise), drugs with purely bactericidal action are less effective at suppressing the emergence of secondary mutations than are drugs with purely bacteriostatic action (**Figure 5E**).

**Index of supplementary files**

**Supplementary File S1 – MATLAB code package containing the code written for this study.** This file contains scripts that we used to implement our model, to analyze data, and to generate simulation data for all main text and supplementary figures. Documentation detailing how to use the software is included in each script of the code package.

**Supplementary File S2 – Parameters for a set of six drugs with different mechanisms of action.** The parameters *α_G_* and *α_D_* describe the steepness of the growth and death rate functions, respectively, around the inflection point. The parameters *γ_G_* and *γ_D_* describe the inflection points of the growth and death rate functions (see **Supplementary Figure S1**). Bacteriostatic potency refers to the magnitude of growth rate decline at saturating concentrations of drug; a value of 1 indicates that that growth rate declines to zero in saturating concentrations of drug (*G*[*i* = *N*] = 0), and a value of 0 indicates that growth rate is unaffected by drug concentration (*G*[*i*] = *G_0_* for all *i*). Bactericidal potency refers to the magnitude of death rate increase at saturating conditions of drug; a value of 1 indicates that death rate increases to maximum in saturating concentrations of drug (*D*[*i* = *N*] = *D_N_* > *D_0_*), and a value of 0 indicates that death rate is unaffected by drug concentration (*D*[*i*] = *D_0_* for all *i*). All other parameters (including drug-target binding rate *k_F_*, drug-target unbinding rate *k_R_*, and target number *N*) are identical for all drugs in the set.

**Supporting Data File S1 – Experimental data for the ciprofloxacin time-kill curve experiment represented in Figure 2A and Supplementary Figure S6.**

**Supporting Data File S2 – Experimentally-measured minimum inhibitory concentrations (MICs) for ciprofloxacin against *Escherichia coli* represented in Figure 2B.** We collated this list of experimentally-measured MICs from the literature; study sources are given in the file.

**Supporting Data File S3 – Model calibrations obtained via simulated annealing.** Starting and ending values for all model parameters are given for each iteration of the model fitting procedure described in **Methods,** *Model calibration via simulated annealing*.

**Supporting Data File S4 – Experimental pharmacodynamic curve data represented in Supplementary Figure S4.** We generated these data by calculating the net growth rates of bacterial populations at each drug concentration in the experiment detailed in **Supporting Data File S1**.

**Supporting Data File S5 – Experimental data for the ampicillin time-kill curve experiment represented in Supplementary Figure S7.**

**References for supplementary material**

[1] J.R. Wiśniewski, D. Rakus, Quantitative analysis of the Escherichia coli proteome, Data Brief 1 (2014) 7-11.

[2] P. Wang, L. Robert, J. Pelletier, W.L. Dang, F. Taddei, A. Wright, S. Jun, Robust growth of Escherichia coli, Curr. Biol. 20(12) (2010) 1099-1103.

[3] M. Terrak, T.K. Ghosh, J. van Heijenoort, J. Van Beeumen, M. Lampilas, J. Aszodi, J.A. Ayala, J.M. Ghuysen, M. Nguyen-Distèche, The catalytic, glycosyl transferase and acyl transferase modules of the cell wall peptidoglycan-polymerizing penicillin-binding protein 1b of Escherichia coli, Mol. Microbiol. 34(2) (1999) 350-364.

[4] P. Schulz zur Wiesch, J. Engelstädter, S. Bonhoeffer, Compensation of fitness costs and reversibility of antibiotic resistance mutations, Antimicrob. Agents Chemother. 54(5) (2010) 2085-2095.

[5] J.L. Martinez, F. Baquero, Mutation frequencies and antibiotic resistance, Antimicrob. Agents Chemother. 44(7) (2000) 1771-1777.

[6] S. Gagneux, C.D. Long, P.M. Small, T. Van, G.K. Schoolnik, B.J.M. Bohannan, The Competitive Cost of Antibiotic Resistance in Mycobacterium tuberculosis, Science 312(5782) (2006) 1944-1946.

[7] T. Dörr, K. Lewis, M. Vulić, SOS Response Induces Persistence to Fluoroquinolones in Escherichia coli, PLOS Genetics 5(12) (2009) e1000760.

[8] R.R. Regoes, C. Wiuff, R.M. Zappala, K.N. Garner, F. Baquero, B.R. Levin, Pharmacodynamic functions: a multiparameter approach to the design of antibiotic treatment regimens, Antimicrob. Agents Chemother. 48(10) (2004) 3670-3676.

[9] T. Johnson, The approach to mutation-selection balance in an infinite asexual population, and the evolution of mutation rates, Proc. Biol. Sci. 266(1436) (1999) 2389-2397.

[10] F. Clarelli, A. Palmer, B. Singh, M. Storflor, S. Lauksund, T. Cohen, S. Abel, P. Abel zur Wiesch, Drug-target binding quantitatively predicts optimal antibiotic dose levels in quinolones, PLoS Computational Biology 16(8) (2020) e1008106.

[11] D.R. Greening, Parallel simulated annealing techniques, Physica D: Nonlinear Phenomena 42(1) (1990) 293-306.

[12] P.A.z. Wiesch, F. Clarelli, T. Cohen, Using Chemical Reaction Kinetics to Predict Optimal Antibiotic Treatment Strategies, PLoS Comput. Biol. 13(1) (2017) e1005321.

[13] D. Fange, K. Nilsson, T. Tenson, M. Ehrenberg, Drug efflux pump deficiency and drug target resistance masking in growing bacteria, Proc. Natl. Acad. Sci. U.S.A. 106(20) (2009) 8215-8220.
